# Immuno-Isolation of Pancreatic Islet Allografts Using Pegylated Nanotherapy Leads to Long-Term Normoglycemia in Full MHC Mismatch Recipient Mice

**DOI:** 10.1371/journal.pone.0050265

**Published:** 2012-12-05

**Authors:** Huansheng Dong, Tarek M. Fahmy, Su M. Metcalfe, Steve L. Morton, Xiao Dong, Luca Inverardi, David B. Adams, Wenda Gao, Hongjun Wang

**Affiliations:** 1 Department of Surgery, Medical University of South Carolina, Charleston, South Carolina, United States of America; 2 Department of Biomedical Engineering, Yale University, New Haven, Connecticut, United States of America; 3 Brain Repair Centre, Department of Neurology, University of Cambridge. Cambridge, United Kingdom; 4 National Center for Coastal Ocean Science, Charleston, South Carolina, United States of America; 5 College of Life Science, Qingdao Agricultural University, Qingdao, P.R. China; 6 Diabetes Research Institute, University of Miami Miller School of Medicine, Miami, Florida, United States of America; 7 Antagen Institute for Biomedical Research, Boston, Massachusetts, United States of America; Children's Hospital Boston/Harvard Medical School, United States of America

## Abstract

Two major hurdles need to be surmounted for cell therapy for diabetes: (i) allo-immune rejection of grafted pancreatic islets, or stem/precursor cell-derived insulin-secreting cells; and (ii) continuing auto-immunity against the diabetogenic endogenous target antigen. Nanotherapeutics offer a novel approach to overcome these problems and here we ask if creation of “stealth” islets encapsulated within a thin cage of pegylated material of 100–200 nanometers thick provides a viable option for islet transplantation. The aims of this study were to test islet viability and functionality following encapsulation within the pegylated cage, and functional efficacy *in vivo* in terms of graft-derived control of normoglycemia in diabetic mice. We first demonstrated that pegylation of the islet surface, plus or minus nanoparticles, improved long-term islet viability *in vitro* compared to non-pegylated (naked) control islets. Moreover, pegylation of the islets with nanoparticles was compatible with glucose-stimulated insulin secretion and insulin biogenesis. We next looked for functionality of the created “stealth” DBA/2 (H-2^d^) islets *in vivo* by comparing glycemic profiles across 4 groups of streptozotozin-induced diabetic C57BL/6 (H-2^b^) recipients of (i) naked islets; (ii) pegylated islets; (iii) pegylated islets with nanoparticles (empty); and (iv) pegylated islets with nanoparticles loaded with a cargo of leukemia inhibitory factor (LIF), a factor both promotes adaptive immune tolerance and regulates pancreatic β cell mass. Without any other treatment, normoglycemia was lost after 17 d (+/−7.5 d) in control group. In striking contrast, recipients in groups (ii), (iii), and (iv) showed long-term (>100 d) normoglycemia involving 30%; 43%, and 57% of the recipients in each respective group. In conclusion, construction of “stealth” islets by pegylation-based nanotherapeutics not only supports islet structure and function, but also effectively isolates the islets from immune-mediated destruction. The added value of nanoparticles to deliver immune modulators plus growth factors such as LIF expands the potential of this novel therapeutic approach to cell therapy for diabetes.

## Introduction

Pancreatic β cell transplantation, either in the form of harvested pancreatic islets, or as cells derived from embryonic precursors or following trans-differentiation *in vitro*, has the potential to restore the recipients' ability to respond to blood glucose levels and secrete insulin in a physiological manner [1]. However major problems in achieving this ideal include lack of donor islets available for transplantation; loss of the valuable resource of islets during the harvesting procedure; and loss of islets following transplantation due to immune mediated allo-rejection plus lack of trophic support [2]. Although future advances in regenerative medicine may alleviate the problem of availability, all these issues are compounded by continuing autoimmunity against the diabetogenic endogenous target antigen. Considering the immune aspects, while efforts have been centered on systematic modulation of host immune responses for transplantation tolerance, the converse of strategies focused on direct protection of the allograft itself has not been adequately explored. With the advent of new technologies and especially nano-scale devices and materials, the concept of creating physical barriers combined with therapeutic support of transplanted islets or cell populations becomes a realistic option.

Islet encapsulation using immune-isolation devices to facilitate the transplantation of islets so reducing the need for immunosuppression has been explored [3], [4]. Macro-capsules (encapsulation of the whole islet graft) and micro-capsulation (encapsulation of single islets) are the most common approaches for encapsulation [5], [6]. However, use of agarose- or alginate-based macro- and micro- capsules is problematic on several counts including lack of clinical-grade biocompatible polymers; the physical thickness of the macro-capsules (mm level) that prevents efficient molecular exchange between the cells of the islet and their micro-environment; and the islet death due to hypoxia and subsequent fibrosis [for review, see [7]]. In the field of transfusion medicine, research has shown that surface modification of red blood cell membranes with non-immunogenic materials such as methoxy[polyethylene glycol] (mPEG) could yield antigenically silent (“stealth”) cells [8]. These “stealth” cells exhibit little or no antisera-mediated agglutination or antibody binding, and show markedly decreased immunogenicity. Moreover, for lymphocytes mPEG modification prevented MHC class II-mediated T cell activation in the mixed leukocyte reaction [9] and the pegylation procedure itself has no negative effects on normal cell structure, function, or viability [10]–[12]. Following these findings, attempts to modify the surface of islets with bioreactive chemicals showed that blood-mediated inflammatory responses to the islets can be reduced [13]: furthermore, pegylated islets exhibit prolonged survival in allogeneic hosts without any immunosuppressive treatment [14], whilst a short course of cyclosporine A therapy synergized for even longer survival [15].

Ideally, islet encapsulation with biocompatible materials should exert both isolation and immunomodulation effects by physically isolating islets from inflammatory cytokines and host immune cells, whilst simultaneously delivering immune regulatory factors *plus* supportive growth factors to the islets. The latter point may allow for relatively low numbers of donor islets providing glycemic control, thereby addressing not only the problem of immune-mediated rejection but also the problems of limited islet supply. However, the PEG of the pegylated layer has insufficient rigidity for loading with a therapeutic cargo: therefore we have explored combining pegylation with nanotherapy.

Very recently biodegradable poly(lactic-co-glycolic acid) (PLGA) nanoparticles have been designed to carry therapeutic agents plus surface targeting moieties able to decorate the surface of pegylated islets [16]–[19]. Compared to the traditional immunoisolation and immunoregulation methods, such nanoparticles provide a biodegradable, biocompatible slow release vehicle for paracrine-type delivery of cargo to the targeted cell or islets. PLGA has been used for drug delivery and is approved by FDA based on its biodegradability, biocompatibility, adjustable biodegradation kinetics, mechanical properties, ease of processing, and safety [20], [21]. PLGA undergoes hydrolysis of the ester linkages in the presences of water to produce the naturally occurring monomers lactic acid and glycolic acid. It has been shown that PLGA nanoparticles loaded with leukemia inhibitory factor (LIF) and targeted to CD4+ T lymphocytes reduce the inflammatory immune response *in vivo* by promoting regulatory T cells (Treg) [22]. In addition to promoting immune tolerance via Treg, LIF is also well known to promote islet cell survival and LIF regulates β cell mass [23]–[25]. Using a full mismatch mouse model, here we ask, (i) does construction of “stealth” islets by pegylation decorated with LIF-nano support islet structure and function? and (ii) are such islets able to maintain normoglycemia following transplantation?

## Materials and Methods

### Animals

Male C57BL/6 and DBA/2 mice at 6–8 weeks of age were purchased from the Jackson Laboratory (Bar harbor, ME). All procedures were carried out using animals less than 12 weeks old and protocols were approved by the IACUC committee at Medical University of South Carolina.

### Islet isolation

DBA/2 mice were anesthetized by intraperitoneal injection of ketamine and xylazine. Each pancreas was perfused with collagenase (type V, 0.6 mg/mL, Sigma Aldrich, St. Louis, MO) through the pancreatic ducts. The dissected enzyme-containing pancreas was then incubated in 37°C water bath with constant shaking to release the islets which were isolated by density gradient separation using standard techniques as described [26]. Islet yield was assessed by the dithizone staining (DTZ, Sigma Aldrich, St. Louis, MO) and converted to a standard number of islet equivalents (IEQ) of islets where the diameter was standardized to 150 µm. Islets were cultured *in vitro* in Dulbecco's Modified Eagles Medium (DMEM) containing 10% of fetal bovine serum at 37°C with 5% CO_2_ using normal or low attachment cell culture plates (Corning, Tewksbury, MA).

### Pegylation and nanoparticle attachment to pegylated islets

Pegylation of freshly isolated mouse islets was carried out by incubation in serum-free DMEM containing the EZ-Link Amine-PEG_11_-Biotin (Thermo Scientific, Rockford, IL) at 20 mg/mL at room temperature for 30 min, followed by washing with PBS. Nanoparticle preparation has been described in detail elsewhere [22]. Briefly, avidin-coated PLGA nanoparticles were loaded with a cargo of either fluorescent dye (coumarin-6), or mouse recombinant LIF (Santa Cruz, CA), using a modified water/oil water double emulsion technique. The diameter of PLGA nanoparticles generated was 100±20 nm (mean ± S.D.). For the LIF-nanoparticles the cumulative LIF release was 1000±50 picograms per milligram particles over a 7-day period [22]. Nanoparticle coating of the islets was performed using a two-step method: freshly isolated mouse islets were first pegylated as above: after washing with PBS, the islets were next incubated with the avidin-coated nanoparticles in complete DMEM medium at 37°C for another 30 min. The decorated islets were then washed in PBS to remove unbound nanoparticles.

### Scanning electron microscopy

Islets were preserved with 1% gluteraldehyde and 0.01% osmium tetroxide followed by dehydration using a graded series (10–100%) of ethanol. Samples were placed on an aluminium stub using double stick tape and sputter coated with gold-platinum using a Denton Vacuum Desk II Sputter Unit prior to examination using a JEOL 5600LV SEM.

### Cell viability analysis

Islets in 1 mL of PBS were stained with 100 µL of SytoGreen 13 (25 M, Invitrogen, Carlsbad, CA) and 100 µL ethidium bromide (EB, 50 M, Sigma Aldrich) at room temperature in the dark. Fluorescence vital staining based on membrane integrity was observed under a confocal microscope. Using this method, dead cells are stained red and live cells are green. Percentage of dead cells in total cells was calculated. At least 10 islets were included in each treatment group. Experiments were repeated for at least 3 times.

### Detection of insulin expression using immunohistochemistry

Naked islets or islets coated with PEG plus empty nanoparticles were cultured in DMEM with high glucose in low attachment plates for 1, 7, 14 and 21 days. Islets were fixed in 4% paraformaldehyde and insulin expression was analyzed by staining with the guinea pig anti-insulin polyclonal antibody (Sigma-Aldrich). A phycoerythrin (PE)-labeled anti-guinea pig secondary antibody was used to detect expression of insulin in individual islets.

### Glucose-stimulated insulin secretion (GSIS) assay

Islets that were either (i) naked, (ii) pegylated, or (iii) pegylated plus nano-empty were placed in 100 mm petri dishes overnight, using some 20 islets per dish. The islets were first treated with DMEM-low glucose (2.8 mM) for 1 hr, and then challenged with DMEM high glucose (28 mM) for a second hour. Cell culture medium was collected and the concentration of insulin released into the growth medium was measured using mouse insulin ELISA kit (ALPCO, Salem, NH). Insulin stimulation index (SI) was calculated as: SI = Insulin concentration after 28 mM glucose stimulation/Insulin concentration after 2.8 mM glucose stimulation.

### Islet transplantation

C57BL/6 (H-2^b^) mice were rendered diabetic by one-time injection of streptozotocin (STZ) given intraperitoneally (i.p.) at 225 mg/kg. Five days after STZ administration, mice with two consecutive blood glucose levels exceeding 350 mg/dL were deemed diabetic and used as recipients. Encapsulated DBA/2 islets (500–600 IEQ) were transplanted under the kidney capsule of each recipient: four groups each of 6–7 recipients each received either (i) naked islets. (ii) pegylated islets; (iii) pegylated plus empty-nano islets; or (iv) pegylated plus LIF-nano islets. Islet function was monitored indirectly by measuring blood glucose levels twice per week. Mice with a blood glucose <200 mg/dL were considered normoglycemic. Grafts were deemed to have been rejected when two consecutive glucose levels were >300 mg/dL after a period of primary graft function evidenced by normoglycemia.

### Statistical analyses

Kaplan-Meier survival curves were based on measurements of normoglycemia and performed by using the StatView software and the statistical differences were assessed by the Log-rank test. Values of p<0.05 were considered significant. Survival data are expressed as mean survival time ± standard deviation (MST ± SD). Differences between each treatment group were compared for statistical significance by the Student's *t* test.

## Results

### 1. Encapsulation improves long-term structural integrity of islets in vitro

**Figure 1:**
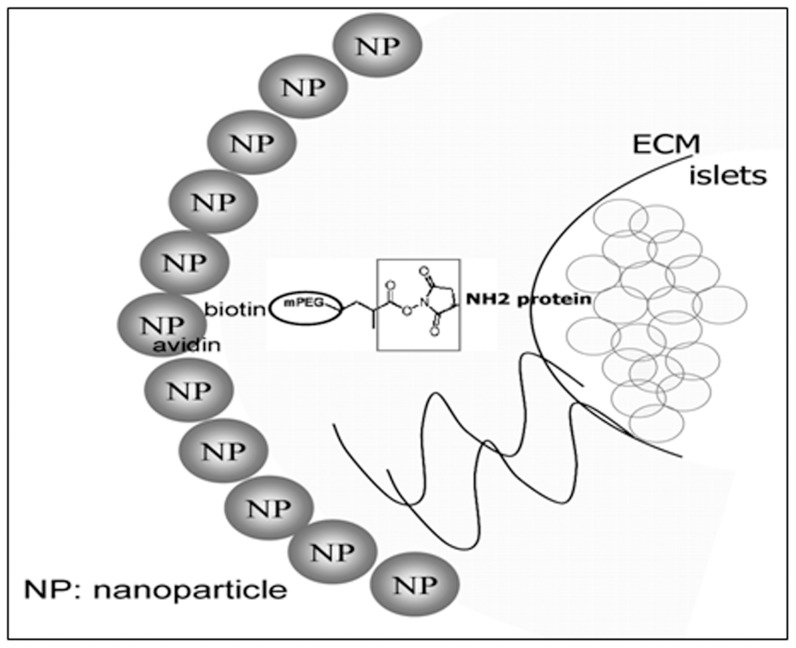
Schematic model of nanoparticles binding to pegylated islet. Avidin groups on the nanoparticle surface mediate nanoparticle attachment to biotinylated PEG that coats the islet.

**Figure 2:**
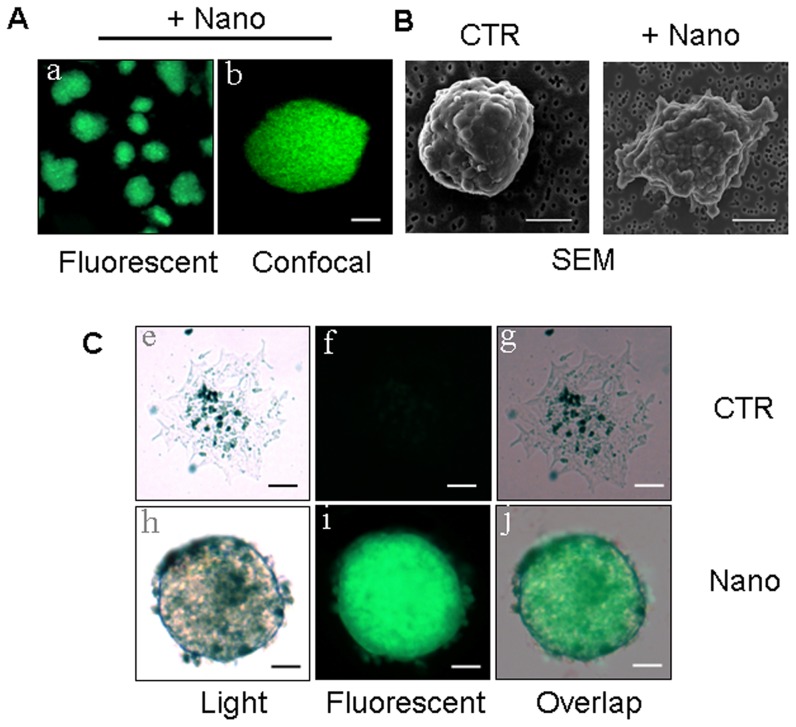
Nanoparticle coating of mouse islets. (A) Islets incubated with PEG plus coumarin-6 (green)-labeled nanoparticles (Nano) observed under fluorescence (left) and confocal (right) microscopes. Scale bar, 50 µm. (B) Naked control islets (CTR), or pegylated islets coated with coumarin-6 labeled nanoparticles (Nano) imaged by SEM immediately after encapsulation. Scale bar, 100 µm. (C) Islets imaged at 21 days post culture: images e–g show naked islets (CTR), images h–j show islets draped with PEG plus coumarin-6-nano (Nano). The naked islets show degradation in marked contrast to the well-preserved nano-pegylated islets. Scale bar, 50 µm.

We first asked, could islets pre-draped with PEG be further decorated with nanoparticles? Freshly isolated mouse islets were incubated with biotin-PEG and then with avidin-nanoparticles loaded with fluorescent dye coumarin-6 (*Fig. 1*.). After washing, the islets were cultured in DMEM with high glucose for 24 h before being examined under fluorescent and confocal microscopes. *Fig. 2* shows that these islets became completely covered with fluorescently labeled nanoparticles (*Fig. 2A*, a). This was confirmed by the Z-stack analysis of confocal microscopy to scan single layers of an islet (*Fig. 2A*, b), thus revealing penetration of the nanoparticles within the islet mass. The interaction of nanoparticles with islet was confirmed using SEM. Here naked islets showed a smooth surface contoured by bumps of individual cells within the islet (*Fig. 2B*, c). In contrast, islets that had been further incubated with coumarin-6-nanoparticles had a rough surface due to the surface bound nanoparticles (*Fig. 2B*, d). These observations demonstrate that avidin-nanoparticles bind to islets coated with biotin-PEG.

We next asked, does encapsulation preserve islet structure? Here we took either naked islets, or islets coated with PEG alone, or islets coated with both PEG plus courmarin-6-nano, and cultured them on normal (high attachment) cell culture plates for up to 21 days. The cultures were monitored daily using fluorescence phase contrast microscopy. In naked islet group, islets lost their coherent islet structure and there was migration of single cells that formed a monolayer. This would be in accordance with loss of basement membrane integrity during islet isolation leading to cell escape from the islets in the absence of pegylation (*Fig. 2C*, e–g). In striking contrast, islets encapsulated with PEG, with or without coumarin-6-nanoparticles, retained an intact islet morphology, as shown for the PEG-nano treated islets in *Fig. 2C*, h–j. Notably, in those islets decorated with both PEG and coumarin-6-nanoparticles, the nanoparticulate coating persisted over the 3 week culture period as indicated by the green fluorescence seen in *Fig. 2C*, h–j. Although at 3 weeks the coumarin-6 dye may not reflect the distribution of the nanoparticles themselves, but rather of diffused drug derived from the nanoparticles, overall the data confirms that nanoparticles do coat the pegylated islets and thereafter release of cargo may continue over 3 weeks when cultured *in vitro*.

### 2. Islet functionality is not impaired by encapsulation

**Figure 3:**
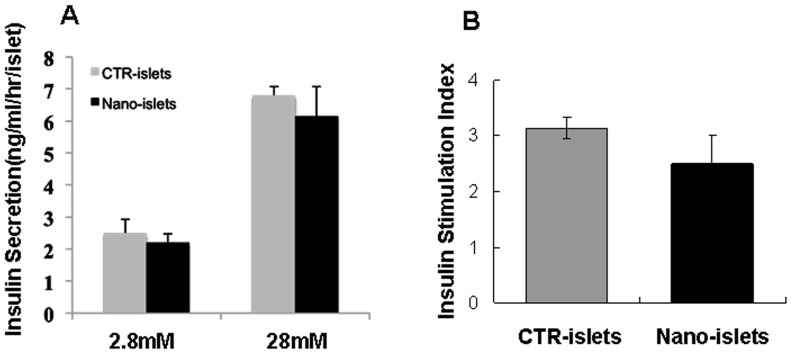
Encapsulation does not affect islet function. (A) Insulin secretion (ng/mL/h/islet) was measured in naked (light grey bars, CTR) and nano-PEG-encapsulated (dark grey bars, Nano) islet cultures cultured overnight after encapsulation stimulated with 2.8 mM, or 28 mM glucose for 24 h. (B) Insulin stimulation index of the naked and nanoparticle-coated islets shown in (A). At least 20 islets were included in each group, and the data represents 3 individual experiments.

Having established that islets can be decorated with a combination of PEG plus nanoparticles with preservation of islet structure, we next determined functional integrity of the encapsulated islets in terms of ability to respond to glucose stimulation. Comparing naked islets with encapsulated islets cultured 1 h in low glucose (2.8 mM) then with 1 h high glucose (28 mM), after overnight in primary culture, we found similar glucose response profiles for insulin release levels (***Fig. 3A***) with correspondingly similar stimulation indices (***Fig. 3B***). We deduced that the encapsulation process did not impair β cell function (i) in sensing glucose change and (ii) in responding to this change with insulin release.

### 3. Prolonged viability of encapsulated islets in vitro

**Figure 4:**
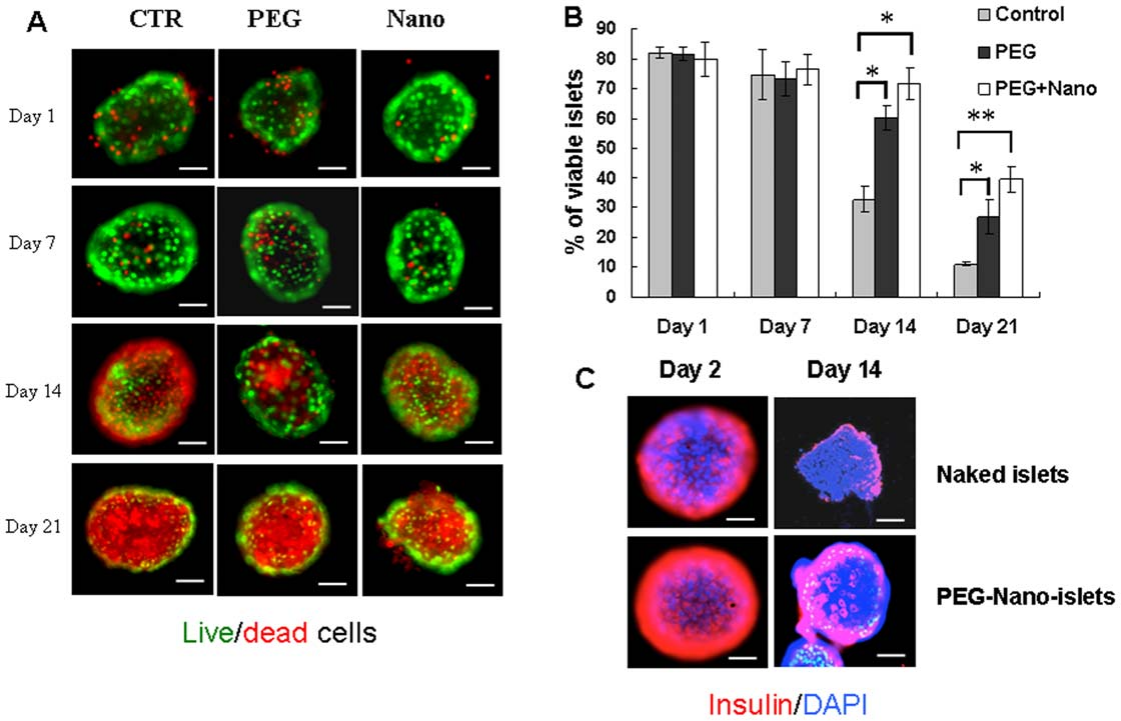
Prolonged viability of encapsulated islets in vitro. (A) Staining of viable (green) *versus* dead (red) cells in cultures of naked islets (CTR), pegylated islets (PEG), or pegylated plus empty-nanoparticle islets (Nano) at 1 d, 7 d, 14 d, and 21 d. (B) Percentages of viable cells in the different groups during culture. (C) Insulin staining in naked (CTR) and PEG-Nano-coated islets at 2 d and 14 d culture: more insulin positive cells were observed in islets encapsulated with nanoparticles (lower panels) compared to naked islets (upper panels). At least 10 islets were included in each group. Red represents insulin staining, blue staining (DAPI) represents nuclear staining in all cells. * p<0.05 and ** p<0.01.

Islet viability *ex vivo* is highly relevant to the potential use of harvested islets for clinical transplantation. We therefore compared naked versus encapsulated islets over a period of 21 d using low attachment conditions to mimic clinical harvest procedure. Three groups, naked islets, pegylated islets, and pegylated islets plus empty nanoparticles, were cultured in DMEM on low attachment cell culture plates. Live and died cells were analyzed at 1, 7, 14 and 21 days after culture using Syto Green and EB staining. ***Fig. 4A*** and B shows that, although viability at 1 d and 7 d was comparable across the three groups at around 75%, there was an unexpected prolongation of long-term viability at both 14 d (∼72%) and 21 d (∼40%) specifically associated with the combined PEG plus nanoparticles. This beneficial effect was significantly greater than pegylation alone at 21 d. The pegylated islets without nanoparticles also showed marked benefits in terms of survival at 14 d (∼60%) and 21 d (∼27%) when compared to the naked islets 14 d (∼33%) and 21 d (∼11%).

Having demonstrated improved long-term viability of islets *ex vivo* afforded by surface pegylation plus nanoparticles, we next confirmed relevance of the surviving islets in terms of their insulin activity. Immunohistochemistry revealed greater positivity in the pegylated plus nanoparticle coated islets compared to naked islets: notably, the difference was clear at 2 d and 14 d (***Fig. 4*** **. *C***). Thus, although viability scores were equivalent at these time points, a difference in the numbers of cells apparently responsive in terms of insulin expression was already present. This may reflect early differential vulnerability of β cells following the trauma of isolation, and/or preferential preservation of β cells following pegylation. Future studies will explore any further protection of β cells gained from attachment of LIF-nanoparticles, or of compound LIF/EGF-nanoparticles, to the pegylated drape, given that LIF is known to support β cells whilst LIF plus EGF synergise in pancreatic β cell differentiation [24].

### 4. Prolonged functionality of encapsulated islets in vivo

**Figure 5:**
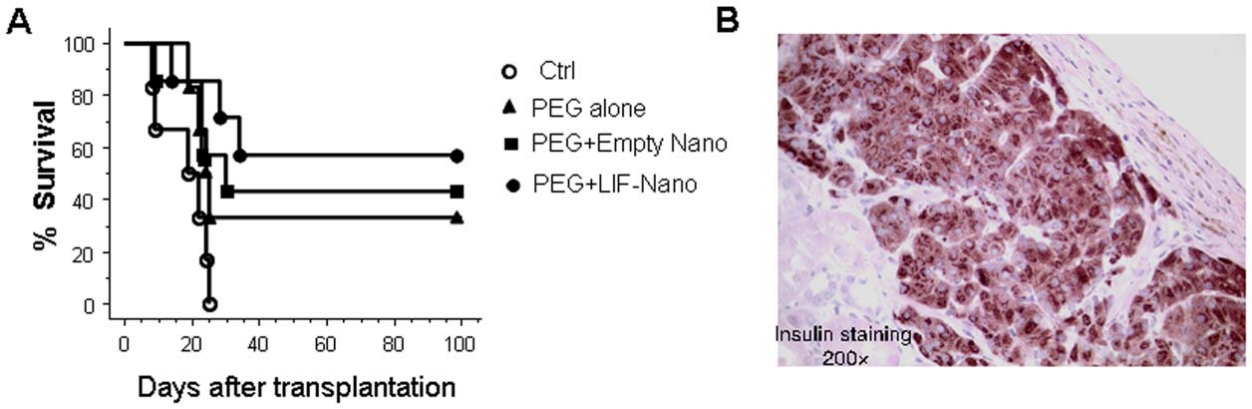
Prolonged functionality of encapsulated islets in vivo. Pancreatic islets from DBA/2 mice were grafted under the kidney capsule of C57BL/6 recipients: the islets were either untreated (Ctrl); or encapsulated in PEG alone (PEG alone); or with PEG decorated with empty nanoparticles (PEG+Empty Nano); or with PEG decorated with LIF-containing nanoparticles (PEG+LIF-Nano). The ability of these grafts to support normoglycemia over 100 d is shown as “% survival” in (A). Histology of grafts taken from recipients showing normoglycemia at 100 d revealed well-preserved β cells containing insulin, as illustrated in (B).

Given the improved viability with continued functionality of β cells in pancreatic islets draped with PEG plus nanoparticles *in vitro*, we next asked, do these pegylated “stealth” islets also show improved functionality *in vivo?* More specifically, could encapsulation protect transplanted islets from the hostile environment of a full MHC mismatched recipient? Using glycaemia as a surrogate indicator of graft function, four groups of six streptozotozin-induced diabetic C57BL/6 recipients were transplanted with islets as follows: (i) naked control islets; (ii) pegylated islets; (iii) pegylated islets with nanoparticles (empty); and (iv) pegylated islets with nanoparticles loaded with a cargo of leukemia inhibitory factor (LIF). LIF was chosen because LIF is known to promote adaptive immune tolerance in addition to LIF playing a key role in regulation of pancreatic β cell mass [24]. The islets were placed under the kidney capsule and no immunosuppressive therapy was given. In group (i) controls, normoglycemia was lost after 17.0 d±7.5 d. In striking contrast, recipients in groups (ii), (iii), and (iv) showed long term (<100 d) normoglycemia involving some 30%, 43% and 57% of the recipients in each respective group. As detailed in ***Fig. 5*** and ***Table 1***, the incidence of long-term normoglycemia in the treated groups (ii)–(iv) was significantly different from the control group (i). This significant therapeutic gain from nanotherapeutic immune-isolation of the islets was interpreted to reflect prolonged islet survival and β cell functionality *in vivo* in the absence of immunosuppression and despite a full MHC mismatch donor/recipient pair combination.

**Table 1:** Long-term normoglycemia derived from pegylated DBA/2 islet grafts in C57BL/6 recipients.

GROUP	N	N>100 d	N<100 d	<100 dMean ± SD (days)	P value vs. control
**(i) Control**	6	0	6	17.0**±**7.5	-
**(ii) Pegylated**	6	2	4	22.5**±**2.6	0.09
**(ii) Pegylated+Empty-Nano**	7	2	5	21.5**±**8.8	0.03
**(iv) Pegylated+LIF-Nano**	7	4	3	27.6**±**6.5	0.003

Grafts were placed under the kidney capsule. No immunosuppressive therapy was given.

“>100 d” indicates number of recipients reaching >100 days normoglycemia.“<100 d” indicates number of recipients failing to reach long-term normoglycemia.

## Discussion

We have constructed “stealth” islets by pegylated nanotherapy wherein the encapsulating pegylated layer is physically linked to nanoparticles for targeted paracrine-type delivery of therapeutic cargo to the immediate microenvironment of the encapsulated islet. The specific aim of this study was firstly to test islet viability and functionality following encapsulation within a pegylated nanoparticle cage, and secondly to test functional efficacy *in vivo* in terms of allograft-derived control of normo-glycaemia in diabetic recipient mice. We demonstrate (i) *in vitro*, prolonged viability and functionality of the “stealth” islets and in particular β cell responsiveness to glucose challenge; and (ii) *in vivo*, prolonged functionality of the “stealth” islet allografts in maintaining normoglycemia in MHC-mismatched diabetic hosts.

Pegylation-based nanotherapeutics of the pancreatic islets significantly reduced the rate of β cell death (***Fig. 4***) – a highly significant point and, even though the *in vitro* model has its limitations in fully mimicking the cell destructive process after transplantation, the data clearly demonstrate that pegylated, nanoparticle decorated islets have superior survival advantages over naked islets. This will underpin new *in vivo* studies aimed at optimising nanotherapeutic cargo for further support of the intra-islet β cell population. Our findings also have immediate relevance to work of others aimed at deriving β cells from stem cells, precursor cells, or by trans-differentiation: the pegylated-nanotherapeutic coat may create cellular micro-environments not only promoting β cell neogenesis, but also thereafter for their “stealth” delivery. For example, we anticipate nanotherapeutic delivery of factors including LIF plus EGF, known to synergise in β cell transdifferentiation from pancreatic exocrine cells [24], [25]. Exocrine pancreas as a source for β cell neogenesis might also be promoted by targeted delivery of inhibitors of the hedgehog signaling pathway based on the recent findings [27].

The ability to reduce the allo-immune response using nano-therapeutics integrated into the pegylated coat of the graft is also a major finding. The added value of targeting immune-modulatory growth factors such as LIF, able to bias allo-responsive T cells towards the Treg lineage [28], becomes especially significant when considering ongoing autoimmunity to endogenous diabetogenic antigen. Although our data is limited to islet allografts under the kidney capsule, and in hosts that are not primed against a diabetogen, the concept holds that shifting differentiation of islet-reactive T cells towards Treg will be beneficial. Importantly, since Treg release LIF upon stimulation by cognate antigen, a self-sustaining state of both immune tolerance plus support for the β cells (via LIF) may arise [29], [30].

In conclusion, nanotherapeutic immune-isolation of grafts creates “stealth” pancreatic islets that show significantly prolonged viability and functionality *in vitro* and also *in vivo*. The long-term normoglycemia in fully mismatched diabetic hosts in the absence of all immunosuppression emphasizes the promise of the “stealth” approach, not only for islet but also for β cell transplantation including for cells generated from stem, precursor, or trans-differentiated, cell sources.
